# Overexpression of the Purple Perilla (*Perilla frutescens* (L.)) *FAD3a* Gene Enhances Salt Tolerance in Soybean

**DOI:** 10.3390/ijms241310533

**Published:** 2023-06-23

**Authors:** Zhan Li, Ying Wang, Lili Yu, Yongzhe Gu, Lijuan Zhang, Jun Wang, Lijuan Qiu

**Affiliations:** 1 MARA Key Laboratory of Sustainable Crop Production in the Middle Reaches of the Yangtze River (Co-Construction by Ministry and Province), College of Agriculture, Yangtze University, Jingzhou 434025, China; zlifreedomair@163.com; 2 National Key Facility for Crop Gene Resources and Genetic Improvement, Institute of Crop Sciences, Chinese Academy of Agricultural Sciences, Beijing 100081, China

**Keywords:** *PfFAD3a*, α-linolenic acid, soybean, salt tolerance

## Abstract

The increasingly serious trend of soil salinization inhibits the normal growth and development of soybeans, leading to reduced yields and a serious threat to global crop production. Microsomal ω-3 fatty acid desaturase encoded by the *FAD3* gene is a plant enzyme that plays a significant role in α-linolenic acid synthesis via regulating the membrane fluidity to better accommodate various abiotic stresses. In this study, *PfFAD3a* was isolated from perilla and overexpressed in soybeans driven by CaMV P35S, and the salt tolerance of transgenic plants was then evaluated. The results showed that overexpression of *PfFAD3a* increased the expression of *PfFAD3a* in both the leaves and seeds of transgenic soybean plants, and α-linolenic acid content also significantly increased; hence, it was shown to significantly enhance the salt tolerance of transgenic plants. Physiological and biochemical analysis showed that overexpression of *PfFAD3a* increased the relative chlorophyll content and PSII maximum photochemical efficiency of transgenic soybean plants under salt stress; meanwhile, a decreased accumulation of MDA, H_2_O_2_, and $O_{2}^{•-}$, increased the activities of superoxide dismutase (SOD), peroxidase (POD), catalase (CAT), and ascorbic acid peroxidase (APX), as well as the production of proline and soluble sugar. In summary, the overexpression of *PfFAD3a* may enhance the salt tolerance in transgenic soybean plants through enhanced membrane fluidity and through the antioxidant capacity induced by C18:3.

## 1. Introduction

Soil salinization is one of the abiotic stresses that severely limits crop yield [1]. Salinization can occur naturally due to groundwater shortage and climate change. Meanwhile, excessive irrigation can also lead to salinization [2]. Currently, more than 20% of arable land is under salt stress, and this problem is still intensifying (FAO Land and Plant Nutrition Management service, http://www.fao.org/nr/aboutnr/nrl/en/ (accessed on 13 July 2019). High salinity can inhibit the growth and development of most crops, and it ultimately results in decreased yields [3].

As an important crop for food and feed, soybean (*Glycine max* (L.) Merr.) is one of the main sources for plant oil and protein. Soybean seeds contain up to about 40% protein and 20% oil [4]. Moreover, soybean seeds also contain bioactive substances such as spermidine, vitamin E, and isoflavones, which are beneficial to human health [5]. It is worth noting that the α-linolenic acid in soybean oil is an important precursor of long chain ω-3 polyunsaturated fatty acids (ω-3 PUFAs) (e.g., EPA, DHA [6]), which are crucial for human growth and development, and which can prevent chronic diseases, especially cardiovascular diseases [7]. However, soybean production is seriously threatened by soil salinization. Soybean is moderately salt-sensitive, and high salt can inhibit the plant growth in the various developmental stages of soybean production, e.g., germination, growth, flowering, and seed filling [8,9,10]. Therefore, breeding toward salt tolerance improvement has become one of the efficient strategies through which to ensure food security. However, traditional hybrid breeding only utilizes limited gene sources and is time-consuming. The transgenic technology can directly modify target crops by introducing exogenous genes into, or altering the expression level of, endogenous genes, thereby providing them with various excellent traits. Therefore, cultivating salt-tolerant soybeans via genetic modification is becoming a research hotspot.

Physiologically, osmotic stress and ion toxicity are caused by high-salt conditions, under which the water absorbance ability from the soil surrounding the plant would be reduced. Under salt stress, the increase in Na^+^ concentration will change the K^+^/Na^+^ ratio, thereby disrupting the cellular ion balance [11]. Salt stress can also trigger oxidative stress. Excessive reactive oxygen species (ROS) attacks damage various cellular components, such as membrane lipids, proteins, and nucleic acids, and these would also consequently cause cell damage [12]. In order to improve the adaptation to salt stress, plants have also evolved salt tolerance mechanisms, such as osmotic regulation and Na^+^/Cl^−^ efflux, as well as compartmentalization, antioxidant systems, and membrane lipid desaturation [13,14]. As a key component of plant membrane lipids, polyunsaturated fatty acids (PUFAs) are the main factor that affect membrane fluidity. There are two main types of polyunsaturated fatty acids in plant membranes, palmitic acid (C16:3) and α-linolenic acid (C18:3), and these determine the membrane fluidity [15,16]. The key step converting linoleic acid (C18:2Δ^9, 12^) into α-linolenic acid (C18:3Δ^9, 12, 15^) is that it is catalyzed by ω-3 fatty acid desaturases (ω-3 FADs). In terms of structure, ω-3 FADs consist of a cytoplasmic domain and several transmembrane domains, where the cytoplasmic domain contains three highly conserved histidine boxes that interact with divalent iron ions and may form the catalytic center of a desaturase [17]. In higher plants, ω-3 FADs were coded by three different genes, including *FAD3*, *FAD7*, and *FAD8*, where *FAD3* encodes microsomal ω-3 FADs, while *FAD7* and *FAD8* encode plastid ω-3 FADs. FAD3 is preferentially located in the endoplasmic reticulum, and its substrates are mainly phosphatidylglycerol (PG) and other phospholipids. In addition, FAD7 and FAD8 are in plastids and mainly act on PG and galactose groups [18]. *FAD7* and *FAD8* are mainly expressed in vegetative organs such as leaves, while *FAD3* is mainly expressed in seeds [19,20]. In addition to regulating the degree of the unsaturation of membrane lipids, α-linolenic acid is also the precursor of oxylipin and jasmonic acid (JA). As we know, JA plays a crucial role in plant growth, development, and the stress response [21]. It is reported that the overexpression of *FAD3*, *FAD7*, and *FAD8* genes can enhance plant resistance to cold, drought, salt, and other abiotic stresses [22]. In summary, using genetically modified technology to improve α-linolenic acid may be an effective way through which to improve the salt tolerance of soybeans.

*Perilla frutescens* (L.) is an annual herbaceous plant that is widely distributed in East Asia and is often used as food or herbal medicine. The content of unsaturated fatty acid in perilla seed oil exceeds 90%, and the content of C18:3 is as high as 52.58%~61.98% [23], which is much higher than that of soybean (3.9%~12.8%) [24]. The *FAD3* in perilla is encoded by two genes, *PfFAD3a* and *PfFAD3b*, whose protein sequence are almost the same, except for one amino acid difference [19]. However, whether this one amino acid difference between *PfFAD3a* and *PfFAD3b* would result in altered physiological characteristics and catalyzation ability remains undetermined. No matter as to how, the high content of C18:3 in perilla sheds light on the possibility that ω-3 polyunsaturated fatty acid content and salt tolerance in soybean could be expected to increase if *PfFAD3* was ectopically expressed.

In this study, *PfFAD3a* was cloned from perilla and overexpressed in soybean. The content of unsaturated fatty acid, salt tolerance, photosynthetic efficiency, and the antioxidant capacity of transgenic plants were evaluated, which provides a gene resource for salt tolerance improvement and lays a foundation for further molecular mechanism analysis.

## 2. Results

### 2.1. Overexpression of PfFAD3a in Soybean

**Figure 1:**
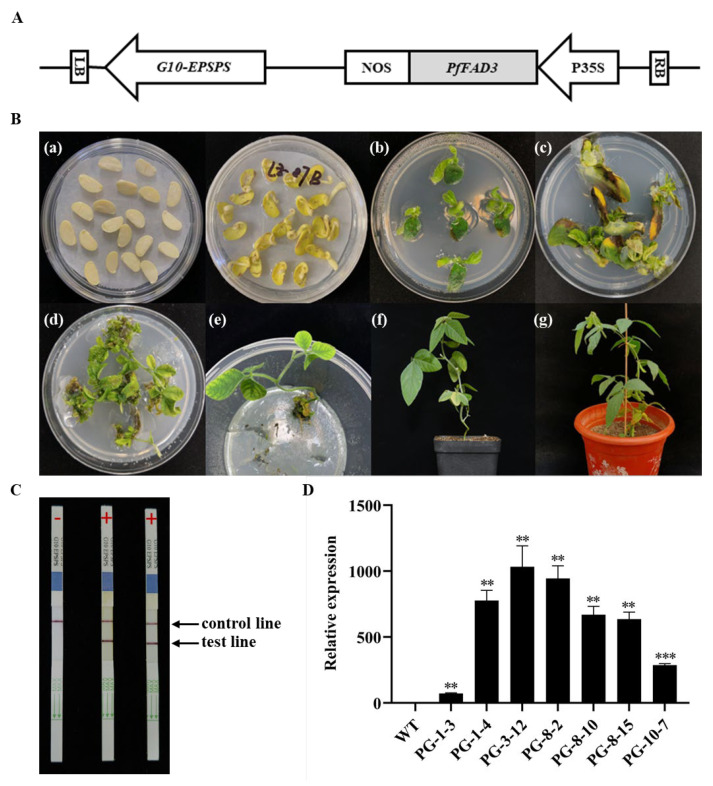
Generation of transgenic soybean plants with an overexpression of *PfFAD3a*. (**A**) The vector structure for soybean genetic transformation. The 1176 bp *PfFAD3a* CDS fragment was cloned into the downstream of the P35S promoter of pCEP01. LB/RB, left/right T-DNA boundary; P35S and NOS, P35S promoter and NOS terminator. The *G10-EPSPS* gene endows the transgenic plants with glyphosate resistance. (**B**) Agrobacterium-mediated soybean genetic transformation procedures: (**a**) the ‘half-seed explant’ (left) after 30 min of Agrobacterium infection and the explant (right) after 5 days of infection; (**b**) recovery for 7 days in a bud induction medium without adding glyphosate; (**c**) incubation in bud induction medium with glyphosate for 21 days; (**d**) shoot extension in a medium supplemented with glyphosate; (**e**) rooting; (**f**) acclimation of transgenic plants; (**g**) transgenic T_0_ plants grown in a greenhouse. (**C**) Transgenic plants identified by G10-EPSPS test strips. ‘+’ and ‘−’ represent positive and negative transgenic plants, respectively. (**D**) The relative expression level of *PfFAD3a* in T_1_ transgenic plants leaves. The bar indicates the SD of three biological replicates. ‘**’ and ‘***’ represent a significance level of 0.01 and 0.001, respectively, and these were determined via Student’s *t*-test when compared with the wild type (WT, blank control).

The CDS of *PfFAD3a* (1176 bp) was first isolated from the cDNA of perilla seeds, and then cloned into pCEP01, which is driven by the CaMV P35S promoter (Figure 1A). Then, *PfFAD3a* was transferred into ‘Jack’ by Agrobacterium, which was mediated with the “half seed method” (Figure 1B). Finally, a total of 12 positive T_0_ transplants were obtained, hereafter designated as PG-1~12 (Figure 1C).

To determine whether *PfFAD3a* is stably expressed in transgenic plants, seven T_1_ single plants from four transgenic lines (PG-1, PG-3, PG-8, and PG-10) were selected for *PfFAD3a* expression level determination via qRT-PCR. The results showed that compared with the wild type (‘Jack’), the *PfFAD3a* gene was successfully expressed in the leaves of all seven T1 individual plants, but its expression level varied between the different lines, with an increase of 71.84~1032.64-fold compared to the blank control (Figure 1D).

### 2.2. Salt Tolerance Evaluation in Transgenic Soybean Plants

**Figure 2:**
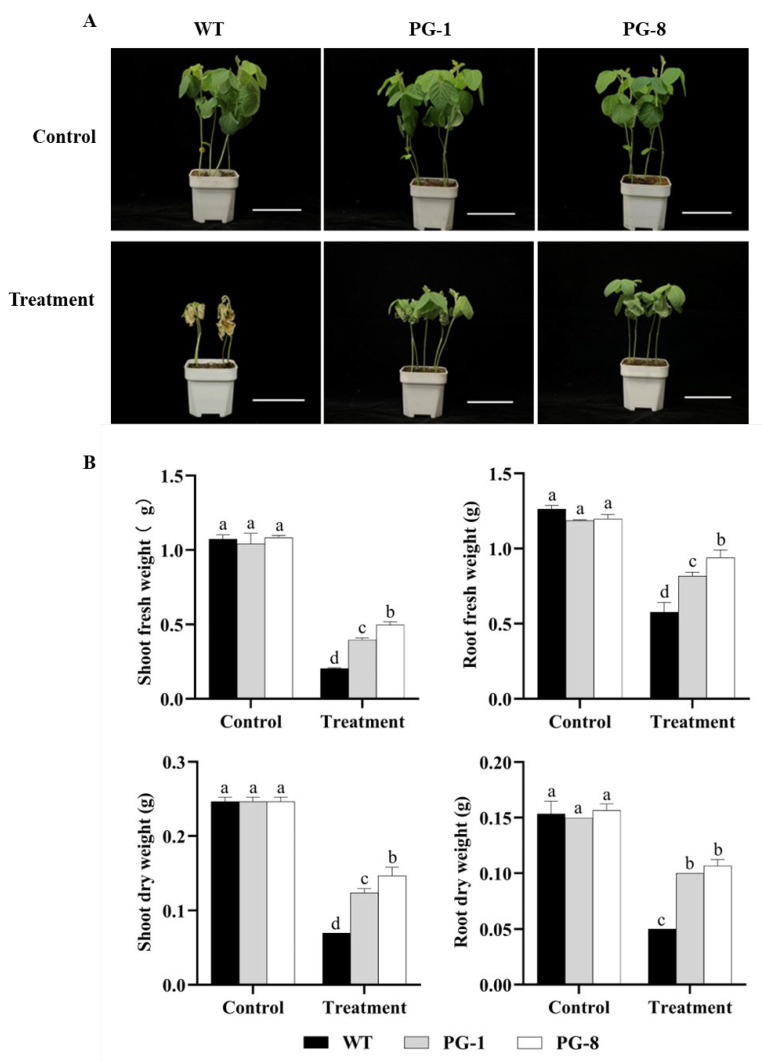
Salt tolerance comparison between the *PfFAD3a*-overexpressed transgenic lines and wild type. (**A**) Phenotypic photos of wild-type and *PfFAD3a*-overexpressed transgenic soybean plants that were treated with or without 150 mM of NaCl for 14 days. Bar = 10 cm. (**B**) The fresh and dry weights of the shoots and roots of wild-type and transgenic plants after 14 days of treatment with or without 150 mM of NaCl. Different lowercase letters above the error bar represent a statistical significance level of *p* < 0.05, as per one-way ANOVA.

To evaluate the salt tolerance capability of plants overexpressing *PfFAD3a*, a 150 mM NaCl solution was applied to 10-day-old T_2_ transgenic soybean seedlings (PG-1 and PG-8) and wild type (‘Jack’) for 14 days. Results showed that both the transgenic plants and wild type were at the V_2_ stage, and there was no difference observed between them in terms of shoot fresh weight (SFW), root fresh weight (RFW), shoot dry weight (SDW), and root dry weight (RDW) in the control (Figure 2A,B). Under salt treatment conditions, the growth of both transgenic soybean seedlings and the wild type was inhibited and stagnated in the V_1_ stage, and all the leaves of the wild-type seedlings withered, including both true leaves and the first trifoliate leaves; however, the first trifoliate leaves of the transgenic soybean seedlings stayed healthy (Figure 2A). In addition, the SFW, RFW, SDW, and RDW of both transgenic soybean seedlings and wild-type seedlings significantly decreased but differed in their extent, to the point that the transgenic seedlings showed less reduction (Figure 2B).

### 2.3. Fatty Acid Composition of PfFAD3a-Overexpressed Plants

**Figure 3:**
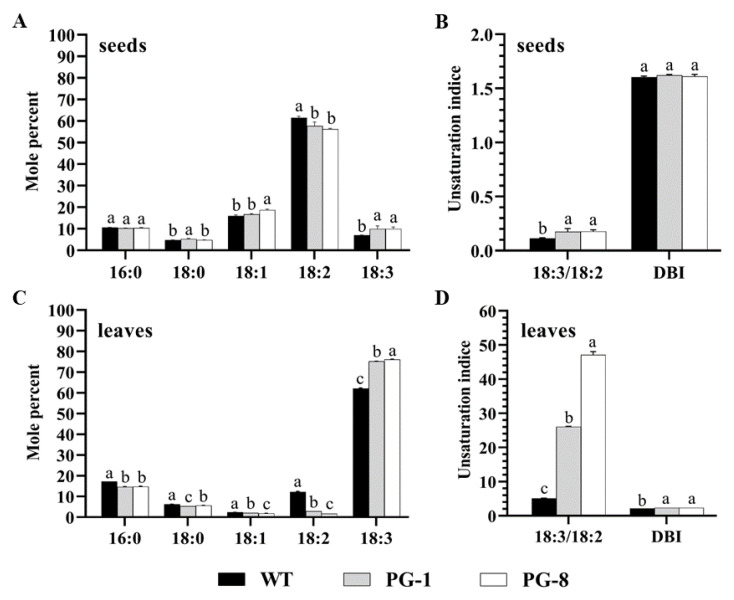
Fatty acid composition of the T_1_ seeds and leaves of plants with *PfFAD3a* overexpression. (**A**) The fatty acid composition of seeds; (**B**) The C18:3/C18:2 ratio and the double bond index (DBI) of seeds, where DBI = [(C18:1%) + (C18:2%) × 2 + (C18:3%) × 3]/100; (**C**) Fatty acid composition of leaves; and (**D**) The C18:3/C18:2 ratio and the double bond index (DBI) of leaves. Different lowercase letters above the error bar represent a statistical significance level of *p* < 0.05, as per one-way ANOVA.

To clarify whether *PfFAD3a* overexpression could influence the fatty acid composition or not, gas chromatography (GC) was used to distinguish each component of fatty acids in both the seeds and leaves of transgenic lines (PG-1 and PG-8). The results showed that the C18:3 content in seeds was significantly increased by 41.64% and 40.51% in PG-1 and PG-8, respectively (*p* < 0.05), while the C18:2 content was decreased significantly (Figure 3A). Similarly, an increment of 21.25% (PG-1) and 22.78% (PG-8) in terms of C18:3, as well in leaves was observed when compared to the wild type (*p* < 0.05), while the content of the other four fatty acids (C16:0, C18:0, C18:1, and C18:2) were all significantly decreased. Among them, C18:2 showed the largest decrease of 76.41% and 86.77% in PG-1 and PG-8, respectively (Figure 3C). With regard to the C18:3/C18:2 ratio, it was significantly higher in both the seeds and leaves of transgenic lines; however, a significant increase, in terms of the double bond index (DBI), was only observed in the transgenic leaves rather than in the seeds (Figure 3B,D).

### 2.4. SPAD and Fv/Fm in PfFAD3a-Overexpressed Plants

**Figure 4:**
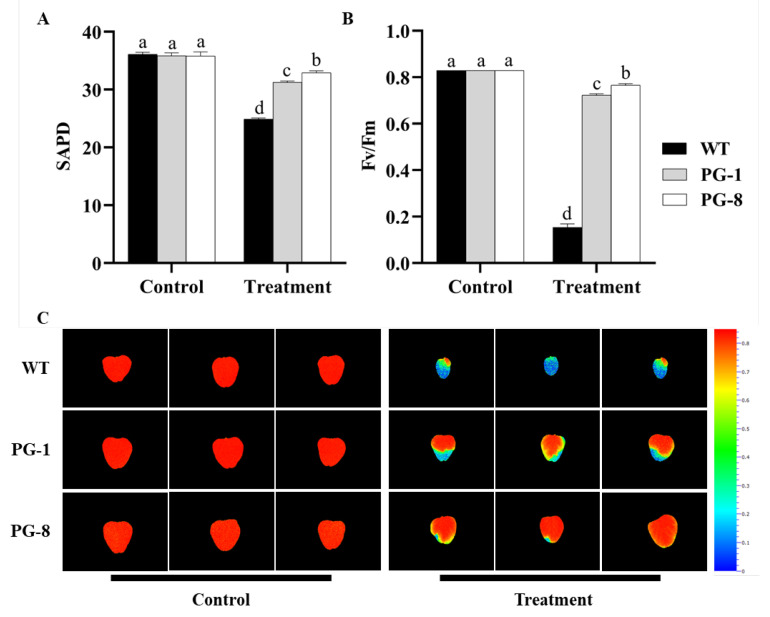
The relative chlorophyll content (SPAD) and PSII photochemical efficiency (Fv/Fm) of plants overexpressing *PfFAD3a* that were under salt stress. (**A**) SPAD values of the true leaves of wild-type and transgenic plants after 7 days of salt treatment or under normal growth conditions. (**B**) The Fv/Fm values of the true leaves of wild-type and transgenic plants after 14 days of salt treatment or under normal growth conditions. (**C**) The Fv/Fm fluorescence images of the true leaves of wild-type and transgenic plants treated with or without salt stress for 14 days. Different lowercase letters above the error bar represent a statistical significance level of *p* < 0.05, as per one-way ANOVA.

To evaluate the impact of the overexpression of *PfFAD3a* on the photosynthetic system, the relative chlorophyll content (SPAD) and maximum photochemical efficiency (Fv/Fm) of photosystem II (PSII) were measured. There was no significant difference in both the SPAD and Fv/Fm between the transgenic and wild-type plants under salt-free conditions (Figure 4A,B). After salt treatment, the SPAD values were significantly reduced by 31.02% (wild type), 12.84% (PG-1), and 8.10% (PG-8), respectively. Moreover, the Fv/Fm values of transgenic plants were significantly higher than that of the wild-type plants by 1.25 and 1.32 times in PG-1 and PG-8, respectively.

### 2.5. Antioxidants in PfFAD3a-Overexpressed Lines

**Figure 5:**
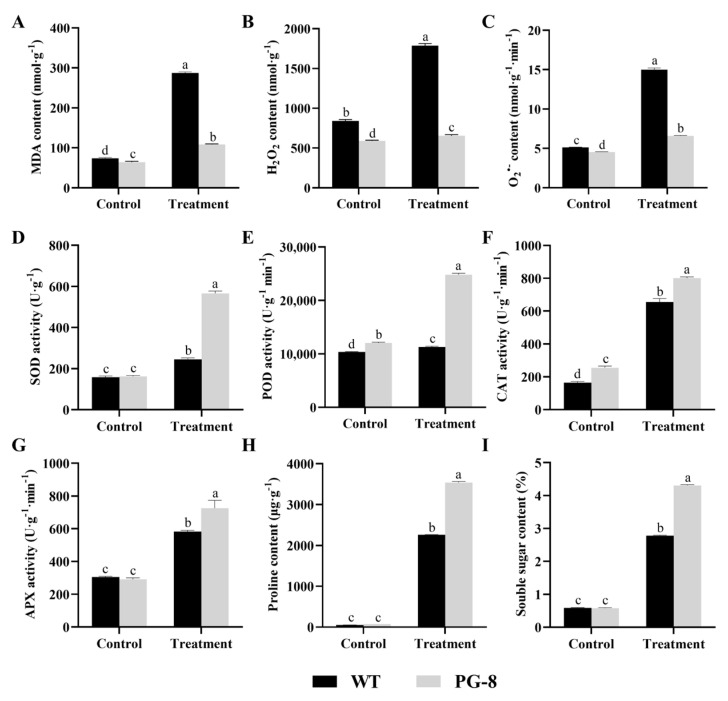
Antioxidant indexes in plants with *PfFAD3a* overexpression under salt stress. (**A**) Malondialdehyde (MDA) content; (**B**) H_2_O_2_ content; (**C**) $O_{2}^{•-}$ content; (**D**) Superoxide dismutase (SOD) activity; (**E**) Peroxidase (POD) activity; (**F**) Catalase (CAT) activity; (**G**) Ascorbate peroxidase (APX) activity; (**H**) Proline content; and (**I**) Soluble sugar content. Different lowercase letters above the error bar represent a statistical significance level of *p* < 0.05, as per one-way ANOVA.

To reveal the effect of the overexpression of *PfFAD3a* on the accumulation of reactive oxygen species (ROS), we measured the contents of MDA, H_2_O_2_, and $O_{2}^{•-}$ in transgenic and wild-type plants. Under normal growth conditions, the content of MDA, H_2_O_2_, and $O_{2}^{•-}$ in transgenic plants is slightly lower than that of wild-type plants. After applying salt stress, the content of MDA, H_2_O_2_, and $O_{2}^{•-}$ in transgenic plants significantly increased by 1.69, 1.11, and 1.46 times, respectively, which was apparently smaller when compared to their extent in the wild-type plants, whose MDA, H_2_O_2_, and $O_{2}^{•-}$ content increased by 3.4, 2.13, and 2.93 times, respectively (Figure 5A–C).

In order to further explore whether *PfFAD3a* overexpression would affect antioxidant enzyme activity or not, the activities of superoxide dismutase (SOD), peroxidase (POD), catalase (CAT), and ascorbate peroxidase (APX) were determined. Results showed that there was no significant difference in the SOD and APX activities between transgenic and wild-type plants under control conditions (Figure 5D,G), but the POD and CAT activities in transgenic plants were significantly increased compared to wild-type plants (Figure 5E,F). Under salt treatment conditions, the activity of the above four antioxidant enzymes significantly increased in both transgenic and wild-type plants, but the increased extent was greater in transgenic plants (Figure 5D–G).

In this study, the content of non-enzymatic antioxidant substances, e.g., free proline and soluble sugar, were also evaluated. Under normal growth conditions, there was no significant difference in proline and soluble sugar content between transgenic and wild-type plants. However, salt stress induced a significant increase in proline and soluble sugar content in both transgenic and wild-type plants, but the contents induced in transgenic plants were significantly higher than those in the wild-type control (Figure 5H,I).

## 3. Discussion

### 3.1. The Salt Tolerance of Soybean Seedlings Could Be Significantly Enhanced via Elevation of α-Linolenic Acid Content

Unsaturated fatty acids (C18:1, C18:2, and C18:3) play a key role in plant stress response [25]. The microsomal enzyme FAD3, and the two plastid enzymes FAD7 and FAD8 are crucial in catalyzing the desaturation from C18:2 to C18:3 [26]. Unsaturated fatty acids are important components of membrane lipids and greatly influence cell membrane fluidity, which is essential for maintaining the normal structure and function of cell membranes [27,28]. It was reported that environmental stresses could induce unsaturated fatty acid content in plant membrane lipids, thus protecting plants from salt and other stresses [29]. The transgenic modification of *FAD3* transcriptional levels in plants is believed to be an effective way through which to alter the levels of trienoic acids in response to environmental stress [30]. In this study, the overexpression of *PfFAD3a* resulted in a significant elevation of α-linolenic acid content and salt tolerance in soybeans (Figure 2 and Figure 3). This finding is consistent with a previous study in which the overexpression of *FAD3* from *Solanum lycopersicum* L. (*LeFAD3*) in tomato eliminated the excess ROS by increasing the level of C18:3; hence, it promoted PSII repairment and the enhanced salt tolerance of transgenic plants during the seedling stage [31,32,33]. In addition, it was reported that overexpression of the *FAD* gene in plants could increase the content of unsaturated fatty acids in transgenic plants and could enhance the resistance of transgenic plants to not only salt, but also to drought and low temperature [16,34,35]. This was possibly because the α-linolenic acid content increased the degree of unsaturation in the membrane lipid and enhanced the fluidity, thus reducing the damage of high salt to the membrane [36].

### 3.2. Photosynthetic Activity Was Enhanced by the Overexpression of PfFAD3a under Salt Stress Conditions

The photosynthetic membrane accommodates photosynthetic pigment protein complexes and electron transfer chains [37]. When plants are subjected to abiotic stresses, the integrity and fluidity of the photosynthetic membrane are easily damaged, which hampers the normal structure and function of the photosynthetic devices [38,39]. The structure and fluidity of the membrane are influenced by lipid composition and the extent of desaturation [28]. Chlorophyll content is highly sensitive to salt stress, and it has been shown that a decrease in chlorophyll content usually occurs under salt stress [40,41,42]. This study demonstrated that salt stress resulted in a decrease in the relative chlorophyll content of both wild-type and transgenic plants (Figure 4A). Salt stress can cause an increase in the concentration of ROS, such as H_2_O_2_ and $O_{2}^{•-}$, consequently resulting in chloroplast membrane peroxidation, which may be one of the main reasons for the decrease in chlorophyll content under salt stress [43]. In this study, the relative chlorophyll content of transgenic plants under salt stress decreased to a lesser extent than that of wild-type plants. This is possibly because the overexpression of *PfFAD3a* augmented the activity of antioxidant enzymes, thereby reducing the oxidative attack of ROS on membranes, photosynthetic devices, and chlorophyll molecules.

PSII is believed to play a crucial role in the response of plant photosynthesis to environmental stresses [44]. In this study, salt stress caused a significant decrease in the Fv/Fm of both wild-type and transgenic plants (Figure 4B,C). Consistently, Yan et al. [45] have reported that after 9 days of treatment with 300 mM of NaCl, the Fv/Fm decreased in both wild-type salt-tolerant and cultivated soybeans. This is probably due to the inhibition of PSII, which disrupts the antenna pigments and limits electron transfer from PSII to PSI, as well as finally causes the degradation of chlorophyll [46,47]. A smaller decrease in the Fv/Fm of transgenic plants under salt stress was observed in this study when compared to wild-type plants, suggesting that the overexpression of *PfFAD3a* weakened the photoinhibition of transgenic plants under salt stress. This is confirmed by a study in *Suaeda salsa*, in which the increase in unsaturated fatty acids in the membrane lipids protected the PSII from damage [48]. Similarly, the combination of light and fatty acid desaturation was proven to be one of the most effective ways through which to protect the photosynthetic devices in synechococcus [49]. The D1 protein, located in the reaction center of PSII, is the most sensitive target in the PSII injury process [50]. When PSII is inhibited by light, the D1 protein at its reaction center continuously vacillates between damage and repair [51]. Therefore, the alleviation of photoinhibition in transgenic lines under salt stress may be explained by the augment of C18:3, which enhances the unsaturation degree of membrane lipids and hence promotes the repair of the D1 protein in the reaction center.

### 3.3. Antioxidative Capacity Was Enhanced via Elevation of C18:3 under Salt Stress

In this study, the activities of SOD, POD, CAT, and APX in transgenic and wild-type plants increased under salt stress (Figure 5D–G), which is consistent with previous research results [52,53,54]. However, Sui et al. [55] found that the activities of antioxidant enzymes such as SOD and APX in peanuts decreased under 250 mM of NaCl treatment. This may be due to the severe damage caused by the high salt treatment intensity to peanut seedlings, which made them unable to resist salt stress by increasing enzyme activity.

Salt stress results in the accumulation of ROS (H_2_O_2_ and $O_{2}^{•-}$ etc.) in cells, which could destroy lipids, proteins, and nucleic acids. MDA is the main product of membrane lipid peroxidation and is commonly used to indicate the degree of oxidative stress and membrane structural integrity in plants [56]. Under unstressed conditions, the content of H_2_O_2_, $O_{2}^{•-}$, and MDA in transgenic plants was significantly lower than those in wild-type plants. When salt stress was applied, the content of these molecules in transgenic plants decreased more significantly than in wild-type plants (Figure 5A–C), which is consistent with the study by Shi et al. [36]. However, the mechanism by which the overexpressed *PfFAD3a* enhanced antioxidant enzyme activity and reduced H_2_O_2_, $O_{2}^{•-}$, and MDA content has not yet been elucidated. Singh et al. [57] revealed that the overexpression of *GmFAD3A* in soybeans can simultaneously improve the salt and drought tolerance of transgenic plants. Not only is the content of C18:3 increased in transgenic plants, but the content of JA is also, suggesting a significant role of JA in the soybean salt stress response. Furthermore, its content may also be related to the increment of C18:3 content. Mata Perez et al. [58] investigated the relationship between linolenic acid, oxidative stress, and the ROS signal transduction mediating response to abiotic stress induced by RNA-seq. Compared to the control, some important differentially expressed genes were associated with the JA biosynthesis pathway in *Arabidopsis* cells cultured with linolenic acid, which suggested that linolenic acid regulates the expression of genes related to the abiotic stress response, especially those mediated by ROS signal transduction. This provided us with the possibility that elevated C18:3 might improve antioxidative capacity and could reduce the ROS accumulation in *PfFAD3a*-overexpressed transgenic soybean plants via the JA regulation pathway; however, this needs to be further tested.

The proline and soluble sugar content in wild-type and transgenic plants was also evaluated in this study, and a significant increment was observed when the transgenic and wild-type plants were under salt stress; however, the transgenic lines showed much higher accumulations (Figure 5H,I). Proline and soluble sugars are not only components of plant non-enzymatic antioxidants, but they are also osmotic regulators maintaining cellular osmotic balance [59]. Many plants accumulate proline to accommodate drought and salt stresses [60]. In this study, the *PfFAD3a*-overexpressed plants accumulated more proline, suggesting a role for *PfFAD3a* in promoting the accumulation of non-enzymatic antioxidants that alleviate the oxidative and osmotic stresses induced by salt conditions. This is consistent with the finding that the proline content in both halophytes and glycophytes has been augmented (note that the content is much higher in halophytes when under salt conditions [61]). However, whether hormones such as JA are involved in the induction of non-enzymic antioxidants under stress needs further investigation. Finally, it should be advised that, strictly speaking, the sudden application of a relatively high concentration of a NaCl solution is called salt shock. The effect of gradually increasing the concentration of a NaCl solution on salt tolerance and its related physiological and biochemical indicators is also a problem worth researching [62].

## 4. Materials and Methods

### 4.1. Vector Construction, Transformation, and Transgenic Lines Identification

A length of 1176 bp CDS was cloned from *Perilla frutescens* and was based on cloning primers, which were designed based on the *PfFAD3a* gene sequence deposited in the GeneBank database (accession number: KX880387) (forward primer 5′-TCCCCGGGATGCTTTTCCGGTGC-3′ and reverse primer 5′-AAACTGCAGTGGCAATCAAGAGTCCATCAC-3′). Then, the cloned fragment was directly cloned into pCEP01 between the *SalI* and *XbaI* restriction sites and was driven by the CaMV P35S promoter. Subsequently, the vector was transformed into ‘Jack’ using an *Agrobacterium*-mediated soybean cotyledon node genetic transformation method [63]. For transgenic plant identification, the transcription levels of the *PfFAD3a* gene in the leaves of four T_1_ individual lines (PG-1, PG-3, PG-8, and PG-10) were quantified using qPCR with the forward primer (5′-CCCACCGTTATTCAGCGT-3′) and reverse primer (5′-GCCGGTTTACTGGGATTGGCTC-3′).

### 4.2. Plant Materials, Growth Conditions, and Salt Tolerance Evaluation

Three lines (one wild-type and two transgenic T_1_ lines, PG-1 and PG-8) were planted in vermiculite with nine plants in each pot. The positive seedlings after 10 days of germination were screened out using genetically modified organization testing G10-EPSPS strips (Cat No. AA1132-LS, YouLong Biotech Ltd., Shanghai, China). The transgenic and wild-type seedlings had relatively consistent growth states and were treated with a NaCl solution (150 mM) or water (control) every 2 days for 2 weeks (14 days). Physiological evaluation was performed after a 7- or 14-day treatment. All plants were grown in the incubator under 12 h/12 h light/darkness with a light intensity of 500 μM·m^−2^·s^−1^, as well as a daytime/nighttime temperature of 26 °C/22 °C and a humidity of 70%.

### 4.3. Phenotype and Biomass Measurement

Plants of both the transgenic lines and wild type were phenotyped after 14 days of cultivation under salt stress or normal growth conditions. The fresh weights of the roots and shoots from the wild-type and *PfFAD3a*-overexpressed soybean plants were scored after separation and were cleaned carefully with tap water. The dry weight (biomass) of each plant was recorded after the roots and shoots were dried in an oven at 65 °C for 72 h to a constant weight. Each experiment was performed for three biological replicates, and for each biological replicate a total of 18 seedlings were tested (three lines and three seedlings per line).

### 4.4. Determination of Fatty Acid Composition

The total fatty acid of the leaves and seeds from wild-type and T_1_ transgenic lines (PG-1 and PG-8) was extracted by the hydrolysis-extraction variant of the internal standard method. The fatty acid composition was determined, according to the National food safety standard-Determination of fatty acid (GB 5009. 168-2016) [64], using the gas chromatography (GC) analyzer GC9720Plus (Fuli Instruments Ltd., Taizhou, China). The internal standard used was triglyceride undecanoate.

### 4.5. Determination of Relative Chlorophyll Content

The relative chlorophyll content of the leaves in both wild-type and transgenic lines was evaluated after 7 days of salt stress treatment with the chlorophyll content analyzer SPAD-502Plus (Minolta Camera Co., Osaka, Japan). The SPAD value was averaged for each biological replicate.

### 4.6. Determination of PSII Photochemical Efficiency

The maximum photochemical efficiency QY-max (i.e., Fv/Fm) of the Photosystem II (PSII) of the true leaves of the wild-type and transgenic plants was measured after 14 days of salt treatment, and this was achieved using the FluorCam chlorophyll fluorescence imaging system FluorCam 800MF (Photon Systems Instruments Co., Brno, Czech Republic).

### 4.7. Measurement of Antioxidant Indexes

The content of MDA, H_2_O_2_, $O_{2}^{•-}$, and the activity of SOD, POD, CAT, and APX, as well as the content of proline and soluble sugar, were measured by Beijing Codon Biotechnology Co., Ltd. Basically, these antioxidant indexes were all measured by spectrophotometry by monitoring the absorbance of the products produced by different methods of measurement at specific wavelengths. The MDA content was measured by the thiobarbituric acid (TBA) method by monitoring the absorbance of the reddish brown 3, 5, 5-trimethyloxazolidine-2, 4-dione at a wavelength of 532 nm [65]. The content of H_2_O_2_ was detected by the titanium sulfate method, and the absorbance of product was detected at 415 nm [66]. The $O_{2}^{•-}$ content was determined by the hydroxylamine oxidation method, and the absorbance of the produced pink azo dye was detected at 530 nm [67]. The enzyme activity of SOD was detected by the nitrogen-blue tetrazolium (NBT) method, and the absorbance of the bule methyl hydrazone product was detected at a wavelength of 560 nm [68]. The POD enzyme activity was measured by the guaiacol method, and the absorbance change in dark brown product was detected at a wavelength of 470 nm [69]. CAT decomposes H_2_O_2_ into H_2_O and O_2_ based on which activity was measured, and a change in the absorbance of H_2_O_2_ at λ = 240 nm was detected [68]. APX catalyzed the reaction between ascorbic acid (AsA) and H_2_O_2_ based on which activity was measured, and the absorbance change in AsA was detected at 290 nm [70]. The content of proline was detected by the acid ninhydrin method, and the absorbance of red product was detected at 520 nm [71]. The content of soluble sugar was detected by the concentrated sulfuric acid-anthrone method, and the absorbance of product produced by sugar and anthrone was detected at 620 nm [72].

### 4.8. Total RNA Extraction and qPCR Analysis

The total RNA was extracted from the leaves of the wild-type and transgenic lines via the SV Total RNA Isolation System (Cat No. Z3100, Promega, Madison, WI, USA). The concentration and purity of the extracted RNA were detected using NanoDrop 2000 (Thermo Scientific, Waltham, MA, USA). The first strand of cDNA was synthesized using the FastKing RT Kit (Cat No. KR116, Tiangen, Beijing, China), the DNase of which also removed the putative remnants of the genomic DNA. A qPCR (quantitative polymerase chain reaction) was performed with a real-time fluorescence quantitative PCR instrument, i.e., the Applied Biosystems 7500 Real-Time PCR System (Thermo Scientific, Waltham, MA, USA), and by using a ChamQ Universal SYBR qPCR Master Mix (Cat No. Q711-02, Novozan, Nanjing, China). The qPCR reaction system consists of 10 µL of Master Mix, 0.4 µL of forward and reverse primers (10 µM), 2 µL of cDNA as the template, and 7.2 µL of H_2_O. The reaction program was set at pre-denaturation at 95 °C for 30 s, followed by 40 amplification cycles, including denaturation at 95 °C for 10 s, as well as annealing and extension at 60 °C for 30 s. The *GmACTIN* gene was used as an internal reference and relative expression level of *PfFAD3a*, and was calculated using the 2^−ΔΔCt^ method [73].

### 4.9. Statistical Analysis

Statistical analyses were performed using the DPS data processing system 7.0 [74]. Students’ *t*-test was used to identify the differences between two groups, and one-way ANOVA was used for multiple comparisons. All data are expressed in the form of the mean ± standard deviation of the three replicates.

## Data Availability

The data are contained within the article. For other information, please contact the corresponding authors.
